# Treatment of Delirium Tremens

**Published:** 1864-02

**Authors:** 


### MISCELLANEOUS.

### TREATMENT OF DELIRIUM TREMENS.

Surgeon William Hanbury, in an interesting article (*Madras Quarterly Journal,* July, 1863,) on the treatment of Delirium Tremens, states that "during the last few years, the cases which have come under my observation have been successfully treated by the use of stimulants (brandy and porter) in limited quantities, and concentrated nourishment during the first two or three days of the affection, followed at the end of that time by the exhibition of opium in anodyne doses at night. The small amount of that medicine, when thus administered, which generally sufficed to induce curative sleep, seemed to suggest that its use could be dispensed with, and that the disease might be left—as far as this medicine was concerned—to the efforts of nature alone, and accordingly an opportunity was taken advantage of to test by experience how far the supposition would prove correct.

"An old and very dissipated soldier, who had been previously treated in the way just indicated, suffered from delirium tremens twice subsequently, and on each of these occasions the characteristic symptoms subsided under the use of stimulants and nutritious food, chiefly beef-tea and egg-flip. Somewhat later a sergeant, much addicted to drink, was admitted with dysentery, aggravated, if not caused by this military vice. At the end of two days the symptoms of delirium tremens became developed, and the cure was trusted to nature alone, aided by nutrients and stimulants, and again with a favorable result.

"A short time after the occurrence of the last case, I was consulted regarding the condition of a man, of very drunken habits, affected by the disease, and who had taken several large doses of opium prescribed in the usual manner. He was delirious and in imminent danger of sinking. The face was collapsed and bedewed with a cold sweat, the pulse was small, rapid, and feeble, and the hands tremulous; and as some cases of cholera were under treatment in the hospital at the time, the impression suggested itself that he had already reached the collaped stage of that disease. A little consideration, however, of the attending circumstances of the case, left no room to doubt that the prostration was due to the unfavorable action of the opium exhibited, and I recommended that its furthur use should be discontinued, and that brandy and porter, with nutritious diet, should be had recourse to. The effect of this change of treatment was very remarkable, and well calculated to make a deep impression. The pulse rallied, the skin became warm, active diaphoresis succeeded to passive serous exudation. A tranquil manner and calm expression of countenance were substituted for nervous tremour and low delirium; and in about thirty hours after the opium was omitted, he fell into a quiet sleep and awoke, cured, at the end of ten hours.

"The injurious influence of opium, and the sufficiency of the expectant or non '*therapeutic*' treatment to effect a cure, were well demonstrated in this case, and I have been informed by the gentlemen who had first to do with it, that the treatment 'without opium,' was also successful in two instances which have since come under his notice.

"But though examples may thus be adduced to prove that opium can be dispensed with, it may well be supposed, in the absence of more numerous facts bearing upon the subject, that the position of a medical man who adopts an expectant treatment must, for the present, of necessity, be a more or less anxious one."

To illustrate the various and uncertain action of opium in the disease Mr. Hanbury gives an account of three different attacks in the same individual, and remarks that in the first the "remedy had no unfavorable effect when given in a single dose, after the symptoms had continued three days, though it is by no means certain that the sleep which occurred at the end of fifteen hours, was due to the action of the opium. In the second, the moderate use of the medicine brought the disease, as usually happens to a favorable termination. In the third, it utterly failed.

"And in now reviewing the facts, I have no doubt that the injurious influence of opium must be referred to the too early exhibition of the medicine, for we have seen that it was prescribed to allay irritability of stomach two days before symptoms of delirium tremens had appeared at all; and it is by no means certain if its use had been further pressed, that the result might not have proved unfavorable. Again, with regard to the stimulants employed, it seems important to note, and especially for the benefit of those who consider them an essential part of the treatment, that although on the last occasion they were administered from the period of admission, yet the disease showed itself two days subsequently. It would appear indeed that the views of Dr. Pirrie and others, who hold the strange mental aberrations and nervous excitement characteristic of the affection, to be the result of toxæmia affecting chiefly the brain substance, are correct. At first sight, no doubt, it might seem that the access of the disease is the direct effect of the withdrawal of the accustomed stimulus, since it so often shows itself in hospitals, as elsewhere, two or three days after a debauch or course of dissipation; but it must be acknowledged the sequence of events in these instances admits of a different explanation, and resting apparently on physiological grounds. The facts themselves are, moreover, at variance with such a conclusion, for we know that the symptoms often immediately supervene on a state of drunkenness; and Dr. Laycock has shown that the disease may be brought to a successful issue without the use either of opium or stimulants, though the latter would obviously be necessary if the abstinence theory of its etiology were tenable.

"On the whole, then, the result of late inquiry and discussion must be assumed to be a more intimate knowledge of the real nature of the disease. There can no longer exist a doubt that the use of opium at an early period of the affection is not only contraindicated, but that nutrients and rest are more nearly concerned with its successful treatment, than the stimulants with which these remedies have been usually associated. Nor shall we be likely to fall into much error in the event of stimulants being considered necessary in any particular case, if we administer them under the guidance of those general principles which are recognized in the management of other diseases.

"Lastly, with respect to digitalis. It will have been noticed that it acted in the case last detailed, to use a common expression, like a charm, though exhibited at a very critical period of the disease; and were this its invariable effect, the treatment of the affection would doubtless be greatly reduced in simplicity, and many anxieties attending it would be removed. But instances of its unfavorable action have been cited, and it still remains to be shown what are the conditions under which it may be had recourse to with least risk of failure.

"I believe it has hitherto proved most useful when not exhibited at too early a stage of the disease, and it may probably be found, as with opium, that large doses from the first invasion of the symptoms are less safe and effectual than smaller ones given at a later date, and after some time has been allowed for the natural evolution of the disease. Moreover, if it be true as Dr. H. Jones suggests, that digitalis exerts a tonic influence on the heart and increases the contractile force of that organ, so far from being inadmissible in the low state of nervous agitation with muttering delirium verging on coma observed in extreme cases, it should here prove especially applicable. Experience, however, must alone determine this point; but in the meantime, and before resorting to the use of digitalis, it will be considered no more than judicious to adopt means calculated to restore the powers of nature, of a kind somewhat similar to those referred to in the case which has called forth these observations."—*Am. Jour. Medical Sciences.*

